# Identification of Different Age-at-Diagnosis-Based Endotypes and Clinical Phenotypes in a Cohort of Adult Patients Diagnosed with Type 1 Diabetes

**DOI:** 10.3390/jcm15124638

**Published:** 2026-06-15

**Authors:** Pedro J. Pinés-Corrales, María C. López-García, Luz M. López-Jiménez, Antonio J. Moya-Moya, Andrés Ruíz de Assín-Valverde, Marina Jara-Vidal, Marta Gallach-Martínez, Cristina Delicado-Hernández, Pablo Mangas-Mellado

**Affiliations:** 1 Department of Endocrinology and Nutrition, Albacete General Hospital, 02002 Albacete, Spain; lopezjimenezluzmaria97@gmail.com (L.M.L.-J.); marinajaravidal@hotmail.com (M.J.-V.); gallachmarta@gmail.com (M.G.-M.); cristinadelicad@gmail.com (C.D.-H.); pablomangasm@gmail.com (P.M.-M.); 2 Department of Endocrinology and Nutrition, Almansa General Hospital, 02008 Albacete, Spain; maricarmenlopez1b@gmail.com; 3 Department of Endocrinology and Nutrition, Hellín General Hospital, 02400 Albacete, Spain; antoniojose_moya13@hotmail.com; 4 Department of Endocrinology and Nutrition, Villarrobledo General Hospital, 02600 Villarrobledo, Spain; andresruizdevalverde@gmail.com

**Keywords:** diabetes mellitus, type 1, biological endotypes, clinical phenotypes

## Abstract

**Background/Objectives**: Type 1 diabetes (T1D) is a heterogeneous disease in terms of clinical presentation, treatment requirements, and risk of complications. The identification of biological endotypes and clinical phenotypes has been proposed to support precision medicine approaches. We aimed to assess the prevalence and clinical characteristics of age-at-diagnosis-based endotypes, adult-onset phenotypes, and insulin-resistant phenotypes in a real-world cohort of adults with T1D. **Methods**: We conducted a single-center, observational, cross-sectional study including adults (≥18 years) with clinically confirmed T1D under active follow-up. Clinical, metabolic, and treatment-related variables were analyzed across predefined age-at-diagnosis-based endotypes and clinical phenotypes. **Results**: A total of 868 patients were included (median age 49 years; diabetes duration 23 years; age at diagnosis 20 years; 51.5% women). Continuous subcutaneous insulin infusion (CSII) was used by 20.4% of patients, and continuous glucose monitoring (CGM) was used by 95.3%. Mean HbA1c was 7.47%, with a median time in range (TIR) of 63%. The prevalence of age-at-diagnosis-based endotype 1 (ED1) was 11.8%, adult-onset phenotype was 31.3%, and insulin-resistant phenotype was 7.3%. No major differences in glycemic control were observed across age-at-diagnosis-based endotypes. Associations between endotypes and treatment-related variables were largely explained by current age and diabetes duration. In contrast, the adult-onset phenotype was independently associated with lower TIR, higher time above range, lower use of CSII, and greater use of adjunctive therapies. The insulin-resistant phenotype was associated with higher HbA1c, lower TIR, and greater therapeutic complexity. **Conclusions**: Adult T1D shows marked heterogeneity. In this real-world cohort, age-at-diagnosis-based endotypes were not independently associated with major clinical differences after adjustment for current age and diabetes duration. In contrast, adult-onset and insulin-resistant phenotypes identified subgroups with poorer glycemic control and greater therapeutic complexity.

## 1. Introduction

Type 1 diabetes (T1D) is a chronic autoimmune disease characterized by progressive pancreatic β-cell destruction, resulting in little or no endogenous insulin secretion, as reflected by low or undetectable plasma C-peptide levels [1]. Although T1D accounts for approximately 5% of all diabetes cases worldwide, it represents a substantial clinical and public health burden [2]. The estimated global incidence is 15 cases per 100,000 person-years, with a prevalence of 9.5 per 10,000 individuals [3]. Epidemiological studies have shown a progressive increase in T1D incidence across most world regions, highlighting its growing public health impact [4].

Prevention of complications in individuals with T1D extends beyond glycemic control and includes optimal management of cardiovascular risk factors such as hypertension and dyslipidemia [5]. In addition, being overweight or being obese is increasingly common among adults with T1D, with prevalence rates comparable to those observed in the general population [6]. Despite major therapeutic and technological advances, T1D remains associated with excess premature mortality and a substantial burden of chronic complications. Recent European registry studies have shown that cumulative exposure to adverse cardiometabolic risk factors—including elevated HbA1c, hypertension, dyslipidemia, smoking, and being overweight—is associated with progressively higher all-cause and cardiovascular mortality [7,8].

Current evidence suggests that T1D is a heterogeneous disease in terms of clinical presentation, pathophysiology, treatment requirements, and risk of complications and mortality [9]. This heterogeneity has led to the proposal of distinct biological endotypes and clinical phenotypes that may contribute to more individualized approaches to disease management. In 2023, Redondo et al. [10] described biologically distinct T1D endotypes associated with differences in age at diagnosis, pancreatic histopathology, inflammatory infiltration, and β-cell dysfunction. Endotype 1 predominates in children diagnosed before 7 years of age, whereas endotype 2 is more frequently observed in individuals diagnosed during adolescence or adulthood. However, direct characterization of biological endotypes requires immunological and molecular markers that are not routinely available in real-world clinical practice. Therefore, in the present study, endotypes were operationally approximated according to age at diagnosis.

A substantial proportion of T1D cases are diagnosed during adulthood (≥30 years), frequently showing marked clinical and metabolic heterogeneity [11]. This includes individuals with classical insulin-deficient presentations as well as others with slower β-cell decline despite confirmed autoimmunity, commonly referred to as latent autoimmune diabetes in adults (LADA) [12]. In addition, factors such as being overweight, having a sedentary lifestyle, and having a family history of type 2 diabetes may contribute to reduced insulin sensitivity, leading to the concept of “double diabetes”, in which autoimmune diabetes coexists with metabolic features typically associated with type 2 diabetes [13]. More recently, Weston et al. [14] proposed an expanded framework integrating biological endotypes and clinical phenotypes, further supporting the heterogeneity of adult T1D and the potential value of more individualized approaches to classification and management.

Adult-onset autoimmune diabetes is frequently defined in the literature using age thresholds around 30 years, particularly in studies exploring LADA-like phenotypes and adult-onset autoimmune diabetes. In parallel, although no universally accepted definition of an insulin-resistant phenotype in T1D exists, high insulin requirements have been widely used as a pragmatic surrogate marker in real-world studies. In this context, we selected an insulin requirement of >1 IU/kg/day to identify patients with marked insulin resistance features in routine clinical practice.

The aim of this study was to describe the prevalence and clinical associations of operational age-at-diagnosis-based endotype classifications, adult-onset phenotypes, and insulin-resistant phenotypes in a large cohort of adults with T1D, and to explore whether these phenotypes identify clinically relevant differences beyond those explained by current age and diabetes duration.

## 2. Materials and Methods

We conducted a single-center, observational, cross-sectional study at a tertiary care hospital serving a population of over 300,000 inhabitants. To improve the applicability of this real-world cohort to routine clinical practice, all electronic health records (EHRs) corresponding to adults with T1D who attended between 1 January 2020 and 31 December 2024 were reviewed.

The inclusion criteria were that the patient had clinically confirmed T1D, the patient’s age was ≥18 years, and the patient had at least one clinical evaluation between 1 January 2024 and the study review period (1 February 2025 to 31 July 2025).

The diagnosis of T1D was based on the clinical assessment of the treating physician and subsequently confirmed through a detailed review of the electronic health records. Diagnostic classification relied on clinical presentation, disease progression, and available autoimmune data when documented in routine clinical practice. Given the retrospective real-world design of the study and the long disease duration of the cohort, systematic C-peptide and autoantibody data at diagnosis were not consistently available.

The overall cohort description included all adults with clinically confirmed T1D under active follow-up. However, for specific clinical, metabolic, and treatment-related analyses, patients with diabetes of a duration < 6 months and those who had undergone combined kidney–pancreas transplantation were excluded when the corresponding variables were considered not clinically comparable or not adequately interpretable. Therefore, denominators varied across analyses according to data availability and evaluability for each specific variable.

The study sample was derived from consecutive inclusion of all eligible patients and was considered appropriate for descriptive analyses, between-group comparisons, and exploratory multivariable analyses.

Diabetic retinopathy was defined by the presence of non-proliferative or proliferative diabetic retinopathy documented in the electronic health record, and the mean HbA1c was defined as the average of the two most recent HbA1c measurements available before data extraction.

Categorical variables are presented as absolute frequencies and percentages, whereas continuous variables are expressed as mean ± standard deviation (SD) or median and interquartile range (IQR), as appropriate. Distributional assumptions were evaluated using graphical methods and normality testing when appropriate. Homogeneity of variances, distribution asymmetry, and the presence of extreme values were also assessed before selecting parametric or non-parametric tests.

Prevalence estimates and corresponding 95% confidence intervals were calculated using the Wilson method. Group comparisons were performed using Student’s *t* test or the Mann–Whitney U test for two-group comparisons, and one-way ANOVA or the Kruskal–Wallis test for multiple-group comparisons. Categorical variables were compared using Pearson’s chi-square test.

Given the retrospective exploratory design of the study, no formal primary or secondary endpoints were predefined. The analyses focused on glycemic control variables, treatment-related variables, chronic complications, and use of nephro- and cardioprotective therapies.

Exploratory multivariable analyses were conducted using multiple linear regression and binary logistic regression models. Covariate selection was based on clinical plausibility, the previous literature, and statistical considerations. Before multivariable analyses, potential collinearity among independent variables was formally assessed using tolerance values and variance inflation factors (VIF). No clinically relevant multicollinearity was identified (all tolerance values > 0.2 and all VIF values < 5). Model assumptions, including linearity of continuous associations when applicable, influential observations, and overall model adequacy, were evaluated before final model interpretation.

Analyses were performed using available data for each variable, and no imputation of missing data was conducted. Given the exploratory nature of the analyses, no correction for multiple comparisons was applied. Statistical analyses were performed using IBM SPSS Statistics version 28.0.0.0 (University of Castilla-La Mancha license). All tests were two-sided, and *p* values < 0.05 were considered statistically significant.

Operational age-at-diagnosis-based endotypes and clinical phenotypes were defined as follows: age-at-diagnosis-based endotype 1 (ED1), age at diagnosis ≤ 7 years; indeterminate endotype, 8–12 years; and age-at-diagnosis-based endotype 2 (ED2), >12 years. The adult-onset phenotype was defined as age at diagnosis ≥ 30 years. The insulin-resistant phenotype was pragmatically defined as an insulin requirement of >1 IU/kg/day, as a pragmatic surrogate marker of insulin resistance in routine real-world practice. Because these classifications were not mutually exclusive, overlap between categories was possible, and three separate analyses were performed: (1) age-at-diagnosis-based endotypes, (2) adult-onset phenotypes, and (3) insulin-resistant phenotypes.

Generative artificial intelligence tools were used exclusively for language editing and technical assistance. All aspects of study design, variable selection, data analysis, interpretation of results, and manuscript conclusions were performed by the authors, who take full responsibility for the content.

The study was approved by the Research Ethics Committee for Medicinal Products of the Albacete Integrated Care Management (18 July 2024; reference No. 2024-091).

## 3. Results

### 3.1. Patient Characteristics

Between 1 January 2020 and 31 December 2024, a total of 1051 adults with clinically confirmed T1D were identified. Of these, 868 patients (82.6%) remained under active follow-up, whereas 103 (9.8%) were lost to follow-up, 39 (3.7%) had died, and 41 (3.9%) had been transferred to other healthcare areas. Table 1 summarizes the baseline characteristics of the cohort according to follow-up status.

### 3.2. Patients’ Characteristics with Active Follow-Up

Baseline demographic, clinical, metabolic, and treatment-related characteristics of the active follow-up cohort are summarized in Table 2. Overall, the cohort was characterized by long-standing T1D, widespread use of CGM technologies, and a substantial prevalence of being overweight or obese. Most patients were treated with multiple daily insulin injections, whereas approximately one-fifth used CSII. CGM-derived metrics were available for 771 patients and showed overall glycemic control comparable to that reported in the contemporary adult T1D cohorts.

Non-insulin therapies were used in a minority of the patients and were mainly represented by metformin and SGLT2 inhibitors. Chronic diabetic complications, particularly diabetic retinopathy, were common, and a substantial proportion of patients received lipid-lowering or nephro- and cardioprotective therapies.

### 3.3. Prevalence of Age-at-Diagnosis-Based Endotypes, Adult-Onset Phenotype, and Insulin-Resistance Phenotype

The prevalence of age-at-diagnosis-based ED1 was 11.8% (102/867; 95% CI: 9.8–14.1%), whereas the indeterminate endotype and ED2 represented 18.3% (159/867; 95% CI: 15.9–21.1%) and 69.9% (606/867; 95% CI: 66.8–72.9%) of evaluable patients, respectively. One patient had no available age at diabetes diagnosis and was therefore not classified into any age-at-diagnosis-based endotype. The prevalence of the adult-onset phenotype was 31.3% (271/867; 95% CI: 28.3–34.5%), and that of the insulin-resistant phenotype was 7.3% (61/830; 95% CI: 5.8–9.3%).

As expected by definition, all patients with the adult-onset phenotype belonged to ED2, whereas the insulin-resistant phenotype was distributed across all age-at-diagnosis-based endotypes. Specifically, six patients with ED1, 19 patients with the indeterminate endotype, and 36 patients with ED2 fulfilled the criteria for the insulin-resistant phenotype. Among patients with ED2, 21 simultaneously presented both the adult-onset and insulin-resistant phenotypes. A detailed overlap analysis between endotypes and phenotypes is presented in Supplementary Table S1.

### 3.4. Analysis of Demographic, Therapeutic, and Glycemic Control Variables, the Presence of Complications, and the Use of Nephro- and Cardioprotective Medications Across Age-at-Diagnosis-Based Endotypes

Patients with age-at-diagnosis-based ED1 were younger and had a longer diabetes duration, whereas those classified as ED2 were older and had a shorter diabetes duration, reflecting the expected collinearity among current age, age at diagnosis, and diabetes duration (Table 3). Overall glycemic control, including HbA1c and CGM-derived metrics, was comparable across endotype groups.

CSII use differed significantly across groups and was more frequent in ED1, whereas MDI predominated in ED2. However, after adjustment for current age and diabetes duration, the age-at-diagnosis-based endotype was not independently associated with CSII use (Table 4). In contrast, younger current age and a longer diabetes duration remained independently associated with higher odds of CSII use.

Non-insulin therapies, lipid-lowering agents, and nephro- and cardioprotective therapies were more frequently prescribed in ED2. However, after multivariable adjustment, these associations were primarily explained by current age and diabetes duration rather than by endotype classification itself (Table 5 and Table 6).

Diabetic retinopathy was more frequent in patients with an earlier age at diagnosis. However, after adjustment, diabetes duration and mean HbA1c remained independently associated with retinopathy, whereas endotype classification did not (Table 7). No significant differences were observed in albuminuria or impaired glomerular filtration rate.

### 3.5. Analysis of Demographic, Therapeutic, and Glycemic Control Variables, the Presence of Complications, and the Use of Nephro- and Cardioprotective Medications Among Patients with and Without an Adult-Onset Phenotype

Patients with an adult-onset phenotype were older at evaluation and had a shorter diabetes duration than the remaining cohort (Table 8). They showed a less favorable CGM-derived glycemic profile, characterized by lower TIR and higher TAR, despite only modest differences in HbA1c. Effect size estimates with corresponding 95% confidence intervals are provided in Supplementary Table S2.

To further explore these findings, a multivariable linear regression model was constructed using TIR as the dependent variable. After adjustment for current age, BMI, TG/HDL ratio, insulin treatment modality, and mean HbA1c, the adult-onset phenotype remained independently associated with lower TIR (Table 9).

Patients with an adult-onset phenotype were less likely to use CSII, whereas CGM use was similar between groups. In contrast, the use of non-insulin therapies, particularly metformin, as well as lipid-lowering and nephro- and cardioprotective therapies, was more frequent in this subgroup.

Regarding complications, patients with an adult-onset phenotype showed a lower crude prevalence of diabetic retinopathy, probably reflecting their shorter diabetes duration. In multivariable analysis adjusted for diabetes duration, mean HbA1c, and current age, adult-onset phenotype was not independently associated with diabetic retinopathy, whereas diabetes duration and mean HbA1c remained independently associated with retinopathy (Supplementary Table S3).

### 3.6. Analysis of Demographic, Therapeutic, and Glycemic Control Variables, the Presence of Complications, and the Use of Nephro- and Cardioprotective Medications Among Patients with and Without an Insulin-Resistance Phenotype

No significant differences in age at diagnosis, current age, or diabetes duration were observed between patients with and without an insulin-resistant phenotype (Table 10).

Patients with an insulin-resistant phenotype showed poorer glycemic control, with higher HbA1c levels, lower TIR, and higher TAR. TBR was numerically higher in this subgroup, although the difference did not reach statistical significance.

No significant differences were observed in insulin treatment modality or CGM use. However, patients with an insulin-resistant phenotype more frequently received metformin, lipid-lowering therapies, fibrates/omega-3 fatty acids, and nephro- and cardioprotective therapies.

Although differences in diabetic retinopathy, albuminuria, and impaired glomerular filtration rate were not statistically significant, these complications were numerically more frequent in patients with an insulin-resistant phenotype.

## 4. Discussion

Our cohort included a large real-world population of adults with T1D characterized by a long disease duration and widespread use of CGM technologies. These characteristics are consistent with a population at relevant risk of chronic diabetes-related complications and provide a useful framework for evaluating clinical heterogeneity in adult T1D. In addition, the relatively low proportion of patients lost to follow-up or transferred to other healthcare areas supports the robustness of the cohort and its applicability to routine clinical practice [7,8,15,16,17,18,19,20].

The prevalence of the age-at-diagnosis-based ED1 classification was 11.8%, whereas the adult-onset and insulin-resistant phenotypes were identified in 31.3% and 7.3% of the cohort, respectively. These findings further highlight the marked clinical heterogeneity of adult T1D. Direct comparison with previous studies is difficult because of differences in study populations, diagnostic criteria, and phenotype definitions. Nevertheless, the substantial proportion of patients diagnosed after 30 years of age is consistent with recent evidence showing that a considerable number of new T1D cases occur during adulthood [21,22,23,24,25].

This observation may have important clinical implications, since β-cell autoimmunity is not uncommon among adults initially classified as having type 2 diabetes [26,27]. These findings support the need for careful diagnostic evaluation in selected adults with atypical clinical features [21]. Similarly, the frequency of the insulin-resistant phenotype observed in our cohort is consistent with the concept of “double diabetes”, which has been associated with obesity, metabolic syndrome, and an adverse vascular risk profile [28,29].

The prevalence of being overweight or obese in our cohort was substantial and comparable to that reported in other international series of adults with T1D [30,31]. These findings are consistent with the growing recognition of metabolic heterogeneity in adult T1D. The use of diabetes technology was also widespread, with 20.4% of patients using CSII and 95.3% using CGM, figures comparable to or slightly higher than those reported in some contemporary cohorts [32,33]. The use of non-insulin therapies was limited and mainly concentrated in selected subgroups, in line with routine clinical practice and current evidence, which does not support their widespread routine use in T1D [34,35,36].

Overall glycemic control was comparable to that observed in other international cohorts [37]. However, the proportion of patients with TBR >4% and particularly > 10% remains clinically relevant, reflecting persistent exposure to hypoglycemia despite broad use of diabetes technologies [38,39]. These findings highlight the importance of continuing to optimize therapeutic strategies aimed at reducing hypoglycemia risk in adults with T1D.

Regarding the operational age-at-diagnosis-based endotype classification, the observed differences in current age and diabetes duration were expected because of the close relationship among these variables and age at onset itself. Although previous prospective studies such as TEDDY and INNODIA have supported the existence of biologically distinct T1D endotypes [40,41], our study used a pragmatic surrogate classification based exclusively on age at diagnosis. In this adult cohort, no major differences in glycemic control were observed across endotype groups when assessed by HbA1c or CGM-derived metrics. The previous literature in this field is heterogeneous and likely reflects not only potential biological differences but also behavioral, healthcare-related, and adherence-related factors [42,43]. Overall, our findings suggest that age-at-diagnosis-based endotype classifications may have limited ability to independently discriminate clinically relevant glycemic differences in adults with T1D.

Although ED1 was associated with a higher proportion of CSII use in the unadjusted analyses, this association was no longer independently significant after adjustment for current age and diabetes duration. This finding is consistent with previous evidence suggesting that technology uptake in T1D is more strongly influenced by factors such as current age, disease duration, socioeconomic context, and healthcare system organization than by age-at-diagnosis-based classifications alone [44,45,46,47,48,49]. Similarly, the greater use of metformin and other non-insulin therapies in ED2, as well as the progressive increase in lipid-lowering and nephro- and cardioprotective therapies with older age at diagnosis, did not remain independently associated after multivariable adjustment. These observations suggest that the identified differences were largely influenced by current age, diabetes duration, and metabolic characteristics commonly observed in adulthood rather than by the operational endotype classification itself [50,51,52,53,54].

Diabetic retinopathy was more frequent among patients diagnosed at younger ages; however, this association did not remain independently significant after adjustment for diabetes duration and HbA1c. These findings are consistent with previous evidence indicating that cumulative glycemic exposure and longer disease duration are major contributors to retinopathy risk in T1D [55,56,57,58,59,60,61,62]. Similarly, no significant differences between age-at-diagnosis-based endotypes were observed for the albuminuria or impaired glomerular filtration rate. Taken together, our findings suggest that operational endotype classifications based exclusively on age at diagnosis may have limited ability to independently discriminate current complication risk in adults with established T1D.

By contrast, the adult-onset phenotype appeared to identify a clinically differentiated subgroup. These patients were older and had shorter diabetes duration, but showed a less favorable CGM-derived glycemic profile, characterized by a lower TIR and a higher TAR, despite only modest differences in HbA1c. In the multivariable analysis, the adult-onset phenotype remained independently associated with lower TIR, highlighting that CGM-derived metrics may capture clinically relevant differences in glycemic control that are not fully reflected by HbA1c alone. Although the available literature on this issue remains limited [63,64], our findings suggest that adult-onset T1D may represent a clinically distinct subgroup, potentially influenced by differences in residual β-cell function, insulin sensitivity, healthcare-related factors, and therapeutic decision-making.

In addition, patients with the adult-onset phenotype used CSII less frequently and were more commonly treated with metformin and nephro- and cardioprotective therapies. Lower use of diabetes technology may partly explain the less favorable glycemic profile observed in this subgroup. This finding is clinically relevant because current guidelines do not recommend restricting access to CSII or automated insulin delivery systems according to age at diagnosis [65]. The greater use of cardiovascular and renal therapies is also consistent with the higher burden of cardiometabolic risk factors observed in these patients and with previous reports describing a less favorable vascular profile in adults with late-onset autoimmune diabetes [66,67].

Although descriptive analyses showed a lower crude prevalence of diabetic retinopathy in patients with the adult-onset phenotype, this association was no longer significant after adjustment for diabetes duration, HbA1c, and current age. These findings suggest that the lower prevalence of retinopathy observed in the descriptive analyses was largely explained by differences in diabetes duration rather than by an independent protective or adverse effect of the adult-onset phenotype itself.

Regarding the insulin-resistant phenotype, no significant differences were observed in current age, age at diagnosis, or diabetes duration. The cutoff used (>1 IU/kg/day) was intentionally conservative and likely identified a relatively small subgroup with more marked insulin resistance, which may have limited statistical power for some analyses. Nevertheless, this phenotype consistently showed worse glycemic control, characterized by higher HbA1c, lower TIR, and higher TAR, in agreement with previous studies linking insulin resistance to poorer metabolic outcomes in T1D [68,69,70]. The absence of differences in CSII or CGM use suggests that these findings are unlikely to be explained solely by differential access to diabetes technology and may instead reflect underlying metabolic characteristics associated with insulin resistance. In this context, lifestyle interventions remain particularly important, since diabetes technologies may improve insulin delivery and glucose management but may not fully address the underlying insulin-resistant state [71].

Patients with the insulin-resistant phenotype also showed greater use of metformin and nephro- and cardioprotective therapies. Although the evidence regarding metformin in T1D remains heterogeneous and does not support its widespread routine use, its prescription in this subgroup likely reflects attempts to address a more complex metabolic profile [72,73,74]. Similarly, the greater use of lipid-lowering agents and renin–angiotensin system blockers is clinically consistent with previous evidence linking insulin resistance in T1D to obesity, dyslipidemia, hypertension, and chronic kidney disease [13]. Although differences in microvascular complications did not reach statistical significance, the observed trend toward higher frequencies of retinopathy, albuminuria, and renal impairment is consistent with the previous literature [75]. The relatively small size of this subgroup may have limited the ability to detect statistically significant differences in some analyses.

Our study has several important strengths. It includes a large real-world cohort of adults with T1D, with a low proportion of follow-up losses and broad use of CGM technologies. In addition, the study jointly evaluates operational age-at-diagnosis-based endotype classifications and clinically defined phenotypes, providing a comprehensive assessment of heterogeneity in adult T1D. The combined use of HbA1c and CGM-derived metrics allowed a more detailed characterization of glycemic control, while multivariable analyses helped explore associations independent of current age and diabetes duration.

Several limitations of this study should be acknowledged. First, the observational and cross-sectional design precludes causal inference. Second, some subgroup analyses, particularly those involving the insulin-resistant phenotype, may have been limited by the relatively small sample size. Third, the diagnosis of T1D and the operational endotype classification were based on routine clinical practice and age at diagnosis, without systematic immunological, molecular, or C-peptide characterization. Therefore, the proposed classifications should be interpreted as pragmatic surrogate approaches rather than biologically validated endotypes. In addition, the use of electronic health records may have resulted in incomplete or non-uniform data for some variables.

## 5. Conclusions

Our results suggest that, in this cohort of adults with T1D, clinical phenotypes showed more consistent associations with metabolic and therapeutic variables than endotypes defined exclusively by age at diagnosis. In this cohort, age-at-diagnosis-based endotypes were not independently associated with major clinical differences after adjustment for current age and diabetes duration. In contrast, the adult-onset and insulin-resistant phenotypes identified subgroups with less favorable metabolic profiles, greater therapeutic complexity, and a potentially higher risk of complications. These observations support further evaluation of phenotype-based approaches in adults with T1D and generate hypotheses for future longitudinal and interventional studies.

## Figures and Tables

**Table 1:** Clinical characteristics according to follow-up status.

Variable	Active (n = 868)	Deceased (n = 39)	Lost to Follow-Up (n = 103)	Transferred (n = 41)	*p*
Current age, years	49 [36–59]	66 [56–76]	44 [31–52]	34 [29–42]	<0.001
Age at diagnosis, years	20 [12–33]	33 [22–44]	18 [10–30]	18 [9–28]	<0.001
Diabetes duration, years	23 [14–34]	33 [20–43]	20 [11–31]	18 [11–23]	<0.001
Male sex, n (%)	422 (48.6%)	22 (56.4%)	60 (58.3%)	23 (56.1%)	0.189

Notes: Data are presented as median [interquartile range] or n (%). Diabetes duration in the active follow-up group corresponds to 867 patients because one patient with combined kidney–pancreas transplantation under active follow-up was excluded from this variable due to a missing date of diabetes diagnosis.

**Table 2:** Baseline characteristics of adults with T1D under active follow-up.

Variable	Overall Cohort
Patients, n	868
Female sex, n/N (%)	446/868 (51.4)
Current age, years	49 [36–59]
Age at diagnosis, years	20 [12–33]
Diabetes duration, years	23 [14–34]
Body weight, kg (n = 831)	75.6 ± 15.8
BMI, kg/m^2^ (n = 831)	26.3 [23.3–29.2]
Overweight, n/N (%)	328/831 (39.5)
Obesity, n/N (%)	172/831 (20.7)
HbA1c, % (n = 868)	7.47 ± 1.15
HbA1c < 7%, n/N (%)	304/868 (35.0)
HbA1c ≥ 8%, n/N (%)	209/868 (24.1)
CGM use, n/N (%)	827/868 (95.3)
Available CGM metrics, n/N (%)	771/868 (88.8)
TIR 70–180 mg/dL, % (n = 771)	63 [50–75]
TBR < 70 mg/dL, % (n = 771)	2 [1–5]
TAR > 180 mg/dL, % (n = 771)	33 [21–46]
TBR > 4%, n/N (%)	324/771 (42.0)
TBR > 10%, n/N (%)	81/771 (10.5)
Total daily insulin dose, IU/day (n = 837)	45 [33–60]
Insulin dose, IU/kg/day (n = 830)	0.61 [0.49–0.76]
MDI, n/N (%)	665/848 (78.4)
CSII, n/N (%)	173/848 (20.4)
Basal insulin alone, n/N (%)	10/848 (1.2)
Any non-insulin therapy, n/N (%)	111/839 (13.2)
Metformin, n/N (%)	56/839 (6.7)
SGLT2 inhibitors, n/N (%)	56/839 (6.7)
GLP-1 receptor agonists, n/N (%)	18/839 (2.1)
Pioglitazone, n/N (%)	1/839 (0.1)
Diabetic retinopathy, n/N (%)	271/835 (32.5)
Chronic kidney disease, n/N (%)	89/846 (10.5)
Lipid-lowering therapy, n/N (%)	396/840 (47.1)
Fibrates/omega-3 fatty acids, n/N (%)	9/840 (1.0)
ACEi/ARB/MRA therapy, n/N (%)	222/840 (26.4)

Notes: Continuous variables are presented as mean ± SD or median [IQR], as appropriate. Percentages were calculated using available evaluable data for each variable. Diabetes duration in the active follow-up group corresponds to 867 patients because one patient with combined kidney–pancreas transplantation under active follow-up was excluded from this variable due to a missing date of diabetes diagnosis. CGM use refers to patients prescribed or actively using continuous glucose monitoring in routine clinical practice, whereas CGM metrics availability refers to patients with retrievable CGM-derived data during the study period. Diabetic retinopathy was defined as documented non-proliferative or proliferative diabetic retinopathy in the electronic health record; patients classified as “not evaluable” were excluded from the denominator. Chronic kidney disease was defined as urinary albumin-to-creatinine ratio (UACR) ≥30 mg/g and/or estimated glomerular filtration rate (eGFR) <60 mL/min/1.73 m^2^; patients without evaluable renal data were excluded from the denominator. MDI: multiple daily injections; CSII: continuous subcutaneous insulin infusion; CGM: continuous glucose monitoring; TIR: time in range; TBR: time below range; TAR: time above range; ACEi: angiotensin-converting enzyme inhibitors; ARB: angiotensin receptor blockers; MRA: mineralocorticoid receptor antagonists.

**Table 3:** Clinical characteristics, glycemic control, treatment, and complications according to age-at-diagnosis-based endotypes.

Variable	ED1 (n = 102)	Indeterminate ED (n = 159)	ED2 (n = 606)	*p*
**Age and disease duration**				
Age at diagnosis, years	4 [2–6]	11 [9–12]	27 [19–39]	<0.001
Current age, years	32 [24–41]	38 [26–47]	53 [43–62]	<0.001
Diabetes duration, years	28 [20–38]	28 [15–37]	21 [12–32]	<0.001
**Glycemic control**				
Mean HbA1c, %	7.46 ± 1.16	7.59 ± 1.31	7.44 ± 1.10	0.334
Available CGM metrics, n/N (%)	86/102 (84.3)	144/159 (90.6)	541/606 (89.3)	—
TIR, %	65.24 ± 16.84	62.12 ± 17.25	61.87 ± 18.19	0.801
TBR, %	2 [1–4]	2 [1–5]	2 [1–5]	0.839
TAR, %	33.48 ± 17.15	34.31 ± 17.36	34.50 ± 18.30	0.886
**Treatment**				
Any non-insulin therapy, n/N (%)	10/99 (10.1)	9/154 (5.8)	92/586 (15.7)	0.004
Metformin, n/N (%)	2/102 (2.0)	2/159 (1.3)	52/606 (8.6)	<0.001
CSII use, n/N (%)	40/100 (40.0)	47/156 (30.1)	86/592 (14.5)	<0.001
CGM use, n/N (%)	93/100 (93.0)	151/156 (96.8)	563/591 (95.3)	0.377
Lipid-lowering therapy, n/N (%)	26/100 (26)	63/154 (40.9)	307/586 (52.4)	<0.001
ACEi/ARB/MRA therapy, n/N (%)	20/100 (20.0)	30/154 (19.5)	172/586 (29.4)	0.014
**Complications**				
Diabetic retinopathy, n/N (%)	39/100 (39.0)	62/153 (40.5)	170/582 (29.2)	0.031
UACR ≥ 30 mg/g, n/N (%)	10/98 (10.2)	12/153 (7.8)	49/565 (8.7)	0.810
eGFR < 60 mL/min/1.73 m^2^, n/N (%)	3/100 (3.0)	5/157 (3.2)	29/589 (4.9)	0.495

Notes: Data are presented as median [interquartile range], mean ± standard deviation, or n/N (%), as appropriate. Percentages were calculated using available evaluable data for each variable. One patient had no available age at diabetes diagnosis and was therefore not classified into any age-at-diagnosis-based endotype. Mean HbA1c was calculated as the average of the two most recent HbA1c determinations available in the electronic health record. CGM use refers to patients prescribed or actively using continuous glucose monitoring in routine clinical practice, whereas available CGM metrics refer to patients with retrievable CGM-derived data during the study period. Diabetic retinopathy was defined as documented non-proliferative or proliferative diabetic retinopathy in the electronic health record; patients classified as “not evaluable” were excluded from the denominator. Chronic kidney disease was defined as urinary albumin-to-creatinine ratio (UACR) ≥30 mg/g and/or estimated glomerular filtration rate (eGFR) <60 mL/min/1.73 m^2^; patients without evaluable renal data were excluded from the denominator. ED: age-at-diagnosis-based endotype; CSII: continuous subcutaneous insulin infusion; CGM: continuous glucose monitoring; TIR: time in range; TBR: time below range; TAR: time above range; UACR: urinary albumin-to-creatinine ratio; eGFR: estimated glomerular filtration rate; ACEi: angiotensin-converting enzyme inhibitors; ARB: angiotensin receptor blockers; MRA: mineralocorticoid receptor antagonists.

**Table 4:** Multivariable logistic regression analysis of factors associated with CSII use according to age-at-diagnosis-based endotype.

Variable	Adjusted OR	95% CI	*p*
Current age (per year)	0.96	0.94–0.98	<0.001
Diabetes duration (per year)	1.04	1.02–1.07	<0.001
ED1 (vs. indeterminate ED)	1.10	0.62–1.95	0.739
ED2 (vs. indeterminate ED)	0.84	0.50–1.39	0.497

Notes: Dependent variable: CSII use. Patients included in the model: n = 847, and CSII users (events): n = 173. One patient without an available age at diabetes diagnosis was excluded because an age-at-diagnosis-based endotype classification could not be performed. Analyses were restricted to patients with evaluable insulin treatment modality data. Covariates were selected according to clinical plausibility, the previous literature, and statistical considerations. Results are expressed as adjusted odds ratios (OR) with 95% confidence intervals (95% CI). No relevant collinearity affecting model stability was identified. ED: age-at-diagnosis-based endotype; CSII: continuous subcutaneous insulin infusion.

**Table 5:** Multivariable logistic regression analysis of factors associated with lipid-lowering therapy use according to age-at-diagnosis-based endotype.

Variable	Adjusted OR	95% CI	*p*
Current age (per year)	1.09	1.07–1.11	<0.001
Diabetes duration (per year)	1.04	1.02–1.05	<0.001
ED1 (vs. indeterminate ED)	0.54	0.26–1.10	0.091
ED2 (vs. indeterminate ED)	0.75	0.44–1.29	0.301

Notes: Dependent variable: use of statins, ezetimibe, bempedoic acid, or PCSK9 inhibitors. Patients included in the model: n = 840; events = 396. Analyses were restricted to patients with evaluable lipid-lowering therapy data and an available age-at-diagnosis-based endotype classification. One patient without an available age at diabetes diagnosis was excluded because endotype classification could not be performed. Covariates were selected according to clinical plausibility, the previous literature, and statistical considerations. Results are expressed as adjusted odds ratios (OR) with 95% confidence intervals (95% CI). No relevant collinearity affecting model stability was identified. ED: age-at-diagnosis-based endotype; PCSK9: proprotein convertase subtilisin/kexin type 9.

**Table 6:** Multivariable logistic regression analysis of factors associated with nephro- and cardioprotective therapy use according to age-at-diagnosis-based endotype.

Variable	Adjusted OR	95% CI	*p*
Current age (per year)	1.08	1.06–1.10	<0.001
Diabetes duration (per year)	1.03	1.01–1.05	<0.001
ED1 (vs. indeterminate ED)	1.27	0.60–2.69	0.534
ED2 (vs. indeterminate ED)	0.71	0.39–1.28	0.257

Notes: Dependent variable: use of ACE inhibitors, angiotensin receptor blockers, or mineralocorticoid receptor antagonists. Patients included in the model: n = 840; events = 222. Covariates were selected according to clinical plausibility, the previous literature, and statistical considerations. Results are expressed as adjusted odds ratios (OR) with 95% confidence intervals (95% CI). No relevant collinearity affecting model stability was identified. ED: age-at-diagnosis-based endotype.

**Table 7:** Multivariable logistic regression analysis of factors associated with diabetic retinopathy according to age-at-diagnosis-based endotype.

Variable	Adjusted OR	95% CI	*p*
ED1 (vs. indeterminate ED)	0.71	0.37–1.34	0.292
ED2 (vs. indeterminate ED)	0.83	0.52–1.31	0.420
Diabetes duration (per year)	1.13	1.11–1.15	<0.001
Mean HbA1c (per 1%)	1.40	1.20–1.65	<0.001

Notes: Dependent variable: diabetic retinopathy. Patients included in the model: n = 803; retinopathy events = 247. Patients classified as “not evaluable” for diabetic retinopathy were excluded from the analysis. Covariates were selected according to clinical plausibility, the previous literature, and statistical considerations. Results are expressed as adjusted odds ratios (OR) with 95% confidence intervals (95% CI). No relevant collinearity affecting model stability was identified. Diabetic retinopathy was defined as documented non-proliferative or proliferative diabetic retinopathy in the electronic health record. Mean HbA1c was calculated as the average of the two most recent HbA1c determinations available in the electronic health record. ED: age-at-diagnosis-based endotype.

**Table 8:** Clinical characteristics, glycemic control, treatment, and complications according to adult-onset phenotype.

Variable	Adult-Onset Phenotype NO (n = 596)	Adult-Onset Phenotype YES (n = 271)	*p*
**Age and disease duration**			
Age at diagnosis, years	14 [9–21]	40 [34–48]	<0.001
Current age, years	42 [30–52]	58 [51–66]	<0.001
Diabetes duration, years	27 [17–37]	17 [8–24]	<0.001
**Glycemic control**			
Mean HbA1c, %	7.46 ± 1.22	7.50 ± 0.96	0.059
Available CGM metrics, n/N (%)	532/596 (89.3)	239/271 (88.2)	—
TIR, %	63.11 ± 17.14	59.75 ± 19.20	0.017
TBR, %	3.55 ± 4.13	3.70 ± 7.12	0.038
TAR, %	33.36 ± 17.63	36.55 ± 18.61	0.019
**Treatment**			
Any non-insulin therapy, n/N (%)	53/596 (8.9)	58/271 (21.4)	<0.001
Metformin, n/N (%)	16/596 (2.7)	40/271 (14.8)	<0.001
CSII use, n/N (%)	143/596 (24.0)	30/271 (11.1)	<0.001
CGM use, n/N (%)	562/596 (94.3)	245/271 (90.4)	0.036
Lipid-lowering therapy, n/N (%)	235/596 (39.4)	161/271 (59.4)	<0.001
ACEi/ARB/MRA therapy, n/N (%)	131/596 (22.0)	91/271 (33.6)	<0.001
**Complications**			
Diabetic retinopathy, n/N (%)	209/565 (37.0)	62/250 (24.8)	<0.001
UACR ≥ 30 mg/g, n/N (%)	49/571 (8.6)	22/245 (9.0)	0.853
eGFR < 60 mL/min/1.73 m^2^, n/N (%)	18/587 (3.1)	19/259 (7.3)	0.005

Notes: Data are presented as median [interquartile range], mean ± standard deviation, or n/N (%), as appropriate. Percentages were calculated using the denominator shown for each variable. Mean HbA1c was calculated as the average of the two most recent HbA1c determinations available in the electronic health record. CGM use refers to patients recorded as using continuous glucose monitoring in routine clinical practice, whereas available CGM metrics refer to patients with retrievable CGM-derived data during the study period. Diabetic retinopathy was defined as documented non-proliferative or proliferative diabetic retinopathy in the electronic health record; patients recorded as not evaluable were excluded from the denominator. CSII: continuous subcutaneous insulin infusion; CGM: continuous glucose monitoring; TIR: time in range; TBR: time below range; TAR: time above range; UACR: urinary albumin-to-creatinine ratio; eGFR: estimated glomerular filtration rate; ACEi: angiotensin-converting enzyme inhibitors; ARB: angiotensin receptor blockers; MRA: mineralocorticoid receptor antagonists.

**Table 9:** Multivariable linear regression analysis of factors associated with TIR (%) according to adult-onset phenotype.

Variable	Adjusted β	95% CI	*p*
Adult-onset phenotype	−2.50	−4.54 to −0.47	0.016
Current age (per year)	0.11	0.05 to 0.17	<0.001
BMI (kg/m^2^)	−0.09	−0.27 to 0.09	0.319
TG/HDL ratio	−0.12	−0.75 to 0.50	0.696
CSII use	12.34	10.26 to 14.41	<0.001
Mean HbA1c (per 1%)	−10.75	−11.54 to −9.50	<0.001

Notes: Dependent variable: TIR (%). Patients included in the model: n = 706. Adjusted R^2^ = 0.46. Covariates were selected according to clinical plausibility, the previous literature, and statistical considerations. Results are expressed as adjusted non-standardized β coefficients with 95% confidence intervals (95% CI). No relevant collinearity affecting model stability was identified. Mean HbA1c was calculated as the average of the two most recent HbA1c determinations available in the electronic health record. TIR: time in range; BMI: body mass index; TG/HDL: triglyceride-to-high-density lipoprotein cholesterol ratio; CSII: continuous subcutaneous insulin infusion.

**Table 10:** Clinical characteristics, glycemic control, treatment, and complications according to insulin-resistant phenotype.

Variable	Non–Insulin-Resistant Phenotype (n = 769)	Insulin-Resistant Phenotype (n = 61)	*p*
**Age and disease duration**			
Age at diagnosis, years	20 [11–33]	22 [15–35]	0.110
Current age, years	48 [36–58]	52 [35–61]	0.342
Diabetes duration, years	24 [14–34]	20 [12–34]	0.537
**Glycemic control**			
Mean HbA1c, %	7.41 ± 1.10	8.12 ± 1.50	<0.001
Available CGM metrics, n/N (%)	687/770 (89.2)	52/61 (85.2)	—
TIR, %	62.72 ± 17.68	53.75 ± 18.62	<0.001
TBR, %	3.48 ± 5.11	5.11 ± 6.62	0.083
TAR, %	33.81 ± 17.83	41.15 ± 19.03	0.004
**Treatment**			
Any non-insulin therapy, n/N (%)	95/770 (12.4)	13/61 (21.3)	0.072
Metformin, n/N (%)	45/770 (5.9)	10/61 (16.4)	0.003
CSII use, n/N (%)	151/770 (19.6)	13/61 (21.3)	0.742
CGM use, n/N (%)	723/770 (93.9)	56/61 (91.8)	0.668
Lipid-lowering therapy, n/N (%)	340/770 (44.1)	37/61 (60.7)	0.015
Fibrate/omega-3 therapy, n/N (%)	6/770 (0.8)	3/61 (4.9)	0.023
ACEi/ARB/MRA therapy, n/N (%)	167/770 (21.7)	21/61 (34.4)	0.029
**Complications**			
Diabetic retinopathy, n/N (%)	233/732 (31.8)	22/56 (39.3)	0.238
UACR ≥ 30 mg/g, n/N (%)	58/669 (8.7)	8/54 (14.8)	0.117
eGFR < 60 mL/min/1.73 m^2^, n/N (%)	27/670 (4.0)	4/49 (8.2)	0.166

Notes: Data are presented as median [interquartile range], mean ± standard deviation, or n/N (%), as appropriate. Percentages were calculated using the denominator shown for each variable. Mean HbA1c was calculated as the average of the two most recent HbA1c determinations available in the electronic health record. CGM use refers to patients recorded as using continuous glucose monitoring in routine clinical practice, whereas available CGM metrics refer to patients with retrievable CGM-derived data during the study period. The insulin-resistant phenotype was defined as an insulin requirement of >1 IU/kg/day. Diabetic retinopathy was defined as documented non-proliferative or proliferative diabetic retinopathy in the electronic health record; patients recorded as not evaluable were excluded from the denominator. CSII: continuous subcutaneous insulin infusion; CGM: continuous glucose monitoring; TIR: time in range; TBR: time below range; TAR: time above range; UACR: urinary albumin-to-creatinine ratio; eGFR: estimated glomerular filtration rate; ACEi: angiotensin-converting enzyme inhibitors; ARB: angiotensin receptor blockers; MRA: mineralocorticoid receptor antagonists.

## Data Availability

All data analyzed in this study are available from the corresponding author upon request.

## Supplementary Materials

The following supporting information can be downloaded at https://www.mdpi.com/article/10.3390/jcm15124638/s1. Table S1: Overlap between age-at-diagnosis-based endotypes, adult-onset phenotype, and insulin-resistant phenotype. Table S2: Effect size estimates for comparisons according to adult-onset phenotype. Table S3: Multivariable logistic regression analysis of factors associated with diabetic retinopathy according to adult-onset phenotype.

## Abbreviations

The following abbreviations are used in this manuscript:

T1D	Type 1 diabetes
ED1	Age-at-diagnosis-based Endotype 1
CSII	Continuous subcutaneous insulin infusion
CGM	Continuous glucose monitoring
TIR	Time in range
TAR	Time above range
ED2	Age-at-diagnosis-based Endotype 2
LADA	Latent autoimmune diabetes in adults
EHRs	Electronic health records
SD	Standard deviation
IQR	Interquartile range
95% IC	95% confidence intervals
BMI	Body mass index
MDI	Multiple daily injections
TBR	Time below range
UACR	Urinary albumin-to-creatinine ratio
eGFR	Estimated glomerular filtration rate
ACEi	ACE inhibitors
ARB	Angiotensin II receptor blockers
MRA	Mineralocorticoid receptor antagonists
