# Supplementary material for: Identification of Different Age-at-Diagnosis-Based Endotypes and Clinical Phenotypes in a Cohort of Adult Patients Diagnosed with Type 1 Diabetes
Source: J Clin Med. 2026 Jun 15;15(12):4638. doi: 10.3390/jcm15124638 (PMC13301096; doi:10.3390/jcm15124638)
Supplement: Supplementary file 1 [file jcm-15-04638-s001.zip › jcm-4303504-supplementary.pdf]

Article

Identification of Different Age-at-diagnosis-based Endotypes and Clinical Phenotypes in a Cohort of Adult Patients Diagnosed with Type 1 Diabetes

Supplementary Table S1. Overlap between age-at-diagnosis-based endotypes, adult-onset phenotype, and insulin-resistant phenotype

Age-at-diagnosis-based endotype	Total, n	Adult-onset phenotype, n/N (%)	Insulin-resistant phenotype, n/N (%)	Adult-onset + insulin-resistant phenotype, n
ED1	102	0/102 (0.0)	6/98 (6.1)	0
Intermediate ED	159	0/159 (0.0)	19/154 (12.3)	0
ED2	606	271/606 (44.7)	36/578 (6.2)	21
Total evaluable	867	271/867 (31.3)	61/830 (7.3)	21

Notes**:** One patient had no available age at diabetes diagnosis and was therefore not classified into any age-at-diagnosis-based endotype. The adult-onset phenotype was defined as age at diagnosis ≥30 years; therefore, all patients with this phenotype were included within ED2. The insulin-resistant phenotype was defined as an insulin requirement >1 IU/kg/day and could occur across all age-at-diagnosis-based endotypes. Percentages were calculated using available evaluable data for each variable.

Supplementary Table S2. Effect size estimates for comparisons according to adult-onset phenotype

Variable	Effect estimate (95% CI)
Current age	Mean difference: +16 years (95% CI: +14 to +18)
Diabetes duration	Mean difference: −10 years (95% CI: −12 to −8)
Mean HbA1c	Mean difference: +0.04% (95% CI: −0.12 to +0.20)
TIR	Mean difference: −3.36% (95% CI: −5.91 to −0.81)
TBR	Median difference: +0.15% (95% CI: −0.10 to +0.40)
TAR	Mean difference: +3.19% (95% CI: +0.58 to +5.80)
Any non-insulin therapy	OR 2.80 (95% CI: 1.89–4.15)
Metformin use	OR 6.29 (95% CI: 3.55–11.15)
CSII use	OR 0.39 (95% CI: 0.25–0.59)
CGM use	OR 0.57 (95% CI: 0.33–0.98)
Lipid-lowering therapy	OR 2.25 (95% CI: 1.66–3.03)

ACEi/ARB/MRA therapy	OR 1.79 (95% CI: 1.29–2.47)
Diabetic retinopathy	OR 0.56 (95% CI: 0.40–0.78)
UACR ≥30 mg/g	OR 1.05 (95% CI: 0.60–1.83)
eGFR <60 mL/min/1.73m²	OR 2.48 (95% CI: 1.26–4.86)

Notes: Continuous variables are presented as mean or median differences with corresponding 95% confidence intervals (95% CI), whereas categorical variables are presented as odds ratios (OR) with 95% CI. Adult-onset phenotype was defined as age at diagnosis ≥30 years. Percentages and effect estimates were calculated using available evaluable data for each variable. OR: odds ratio; CI: confidence interval; CSII: continuous subcutaneous insulin infusion; CGM: continuous glucose monitoring; TIR: time in range; TBR: time below range; TAR: time above range; UACR: urinary albumin-to-creatinine ratio; eGFR: estimated glomerular filtration rate; ACEi: angiotensin-converting enzyme inhibitors; ARB: angiotensin receptor blockers; MRA: mineralocorticoid receptor antagonists.

Supplementary Table S3. Multivariable logistic regression analysis of factors associated with diabetic retinopathy according to adult-onset phenotype

**Variable**	**Adjusted OR**	**95% CI**	**p**
Adult-onset phenotype	1.50	0.74–3.04	0.257
Diabetes duration, per year	1.13	1.10–1.16	<0.001
Mean HbA1c, per 1%	1.41	1.20–1.66	<0.001
Current age, per year	1.01	0.98–1.03	0.529

Notes: Dependent variable: diabetic retinopathy. Patients included in the model: n = 803; retinopathy events = 269. Patients classified as “not evaluable” for diabetic retinopathy or with missing covariate data were excluded from the analysis. Results are expressed as adjusted odds ratios (OR) with 95% confidence intervals (95% CI). Mean HbA1c was calculated as the average of the two most recent HbA1c determinations available in the electronic health record.
